# Derivation of the equation of isostatic line of compression and splitting force in a bottle-shaped strut

**DOI:** 10.1016/j.mex.2018.07.008

**Published:** 2018-07-27

**Authors:** Aimin Yuan, Maierdan Aikebaier, Shoulong Qian, Hang Dai

**Affiliations:** a Dept. of Civil and Transportation Engineering, Hohai University, China; b College of Civil Engineering, Southeast University, China

**Keywords:** Derivation of the equation, ILC, Splitting force, Bottle-shaped strut, Height-to-width ratios

## Abstract

The reported data deal with the derivation of the equation of isostatic line of compression (ILC) and splitting force in a bottle-shaped strut with different height-to-width ratios. The final data show that the splitting force in a bottle-shaped strut is not only related to the height-to-width ratio (h/b), but also related to the load area ratio.

**Specifications Table**

**Table:** 

Subject area	*Engineering*
More specific subject area	*Bridge Engineering*
Type of data	*Derivation process of equations*
How data was acquired	*Data deduced based on mathematics theories*
Data format	*analyzed*
Experimental factors	*No pretreatment*
Experimental features	*Very brief experimental description*
Data source location	*Nanjing, China*
Data accessibility	*Data is displayed within this article.*

## Value of the data

• The data provide the detailed derivation process of the equation of isostatic line of compression.
• The data provide a formula to calculate the magnitude of the splitting force
• The data provide a formula to determine the location of the resultant splitting force.
• The data serves as a methodological benchmark for further attempts to improve the formula of the splitting force subjected to different geometric and boundary conditions.

## Data

• Considering the different assumptions based on the previous researches, A mathematic and explicit describing the equation of ILCs is established.
• Splitting force formulae for the struts with different load area ratios are obtained.

## Experimental design, materials and methods

### Derivation of the equation of ILCs

**Fig. 1:**
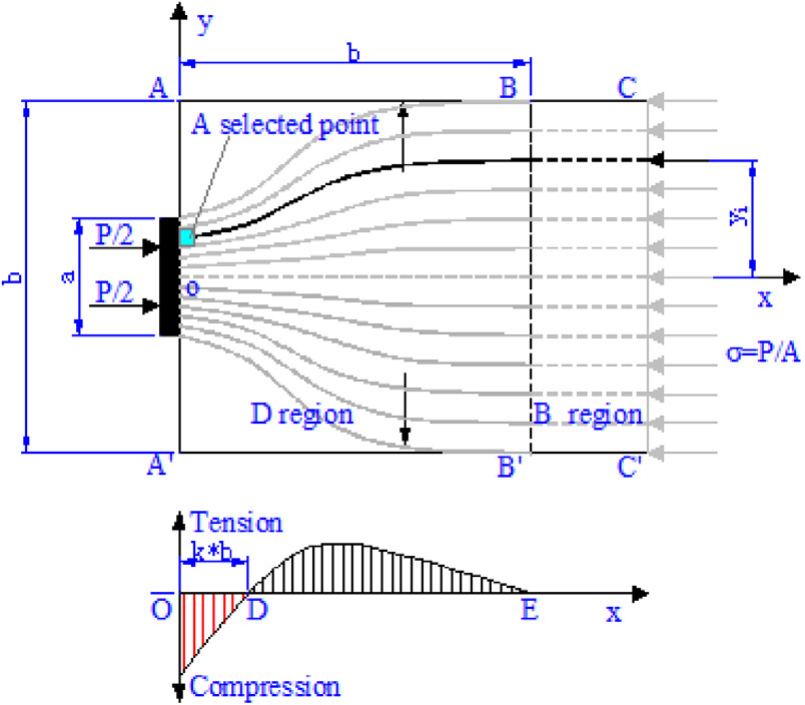
Calculation model for ILC equations.

This datea article refers to the research paper Splitting force of Bottle-shaped Struts with Different Height to Width Ratios (Yuan et al,in press) [1]. In the region of the struts under a concentrated load, the typical dispersion of compression is shown in Fig. 1.

To calculate the transverse stresses, the CDM should be defined as the mathematical model of principal compressive-stress trajectories. Five geometric and physical boundary conditions of the ILCs of the CDM are given as follows.

(1) $y\left|{}_{x=0}=y_{i}\frac{a}{b}\right)$; (2) $y\left|{}_{x=b}=y_{i}\right)$; (3) ${\left(\frac{dy}{dx}\right|}_{x=b}=0$; (4)${\left(\frac{d^{2}y}{dx^{2}}\right|}_{x=b}=0$; (5)${\left(\frac{d^{2}y}{dx^{2}}\right|}_{x=k\cdot b}=0$

The equations of ILCs are assumed to have the polynomials form, given by

(1) $$y=\text{A}x^{4}+\text{B}x^{3}+\text{C}x^{2}+\text{D}x+\text{E}$$

The first and second derivative of Eq. (1) are

(2) $$\frac{dy}{dx}=4\text{A}x^{3}+3\text{B}x^{2}+2\text{C}x+D$$

(3) $$\frac{d^{2}y}{dx^{2}}=12\text{A}x^{2}+6\text{B}x+2\text{C}$$

For ${\left(\frac{dy}{dx}\right|}_{x=b}=0$

(4) $$4\text{A}b^{3}+3\text{B}b^{2}+2\text{C}b+D=0$$

For $y\left|{}_{x=0}=y_{i}\frac{a}{b}\right)$

(5) $$E=y_{i}\cdot \frac{a}{b}$$

For $y\left|{}_{x=b}=y_{i}\right)$

(6) $$\text{A}b^{4}+\text{B}b^{3}+\text{C}b^{2}+Db+y_{i}\frac{a}{b}=y_{i}$$

For ${\left(\frac{d^{2}y}{dx^{2}}\right|}_{x=b}=0$

(7) $$12\text{A}b^{2}+6\text{B}b+2\text{C}=0$$

For ${\left(\frac{d^{2}y}{dx^{2}}\right|}_{x=k\cdot b}=0$

(8) $$12\text{A}k^{2}b^{2}+6\text{B}kb+2\text{C}=0$$

(4) × *b*− (6):

(9) $$3\text{A}b^{4}+2\text{B}b^{3}+\text{C}b^{2}-y_{i}\frac{a}{b}=-y_{i}$$

(8) − (7):

(10) $$\begin{array}{c} 12\text{A}\left(k^{2}-1\right)b^{2}+6\text{B}\left(k-1\right)b=0\to 12\text{A}\left(k+1\right)b^{2}+6\text{B}b=0\\ 2\text{A}\left(k+1\right)b+\text{B}=0 \end{array}$$

(7) × *b*^2^ − (9) × 2:

(11) $$\begin{array}{c} 6\text{A}b^{4}+2\text{B}b^{3}+y_{i}\frac{2a}{b}=2y_{i}\\ 3\text{A}b^{4}+\text{B}b^{3}+y_{i}\frac{a}{b}=y_{i} \end{array}$$

By Substituting Eq. (10) into Eq. (11):

(12) $$\begin{array}{c} 3\text{A}b^{4}-\text{2A}\left(k+1\right)b\cdot b^{3}+y_{i}\frac{a}{b}=y_{i}\\ \text{A}=\left(1-\frac{a}{b}\right)y_{i}/\left(1-2k\right)b^{4} \end{array}$$

(13) $$\text{B}=-2\left(1-\frac{a}{b}\right)y_{i}\cdot \left(k+1\right)\cdot b/\left(1-2k\right)b^{4}$$

By Substituting Eqs. (12) and (13) into Eq. (7):

(14) $$\begin{array}{c} 12\times \frac{\left(1-a/b\right)y_{i}}{\left(1-2k\right)b^{4}}b^{2}+6\times \frac{-2\left(1-a/b\right)y_{i}\left(1+k\right)b}{\left(1-2k\right)b^{4}}\cdot b+2C=0\\ \text{C}=-\frac{6\left(1-\frac{a}{b}\right)b^{2}\cdot y_{i}-6\left(1-\frac{a}{b}\right)b^{2}\cdot y_{i}\left(1+k\right)}{\left(1-2k\right)\cdot b^{4}}=\frac{6\left(1-\frac{a}{b}\right)b^{2}\cdot y_{i}\cdot k}{\left(1-2k\right)\cdot b^{4}} \end{array}$$

By Substituting Eqs. (12), (13), (14) into Eq. (4):

(15) $$\begin{array}{c} D=-\left(4\text{A}b^{3}+3\text{B}b^{2}+2\text{C}b\right)\\ =-\left(4\cdot \frac{\left(1-\frac{a}{b}\right)\cdot y_{i}}{\left(1-2k\right)\cdot b^{4}}\cdot b^{3}+3\cdot \frac{-2\left(1-\frac{a}{b}\right)b\cdot y_{i}\left(1+k\right)}{\left(1-2k\right)\cdot b^{4}}\cdot b^{2}+2\cdot \frac{6\left(1-\frac{a}{b}\right)b\cdot y_{i}\cdot b^{2}k}{\left(1-2k\right)\cdot b^{4}}\cdot b\right)\\ =-\frac{\left[4-6\left(k+1\right)+12k\right]\cdot \left(1-\frac{a}{b}\right)y_{i}}{\left(1-2k\right)b}\\ =-\frac{4-6k-6+12k}{\left(1-2k\right)b}\left(1-\frac{a}{b}\right)y_{i}\\ =-\frac{-2+6k}{\left(1-2k\right)b}\left(1-\frac{a}{b}\right)y_{i}=\frac{2\left(1-3k\right)}{\left(1-2k\right)b}\left(1-\frac{a}{b}\right)y_{i} \end{array}$$

By Substituting A–E into Eq. (1), we have that

(16) $$\begin{array}{c} y=\frac{\left(1-\frac{a}{b}\right)y_{i}}{\left(1-2k\right)b^{4}}x^{4}+\frac{-2\left(1-\frac{a}{b}\right)b\cdot y_{i}\left(1+k\right)}{\left(1-2k\right)b^{4}}x^{3}+\frac{6\left(1-\frac{a}{b}\right)\cdot y_{i}b^{2}k}{\left(1-2k\right)b^{4}}x^{2}+\frac{2\left(1-3k\right)}{\left(1-2k\right)b}\left(1-\frac{a}{b}\right)y_{i}x+\frac{a}{b}y_{i}\\ =\left\{\frac{\left(1-\frac{a}{b}\right)x}{\left(1-2k\right)b}\left[\frac{x^{3}}{b^{3}}-\frac{2\left(1+k\right)}{b^{2}}x^{2}+\frac{6k}{b}x+2\left(1-3k\right)\right]+\frac{a}{b}\right\}\cdot y_{i} \end{array}$$

### Derivation of the transverse stresses

Based on the above results, we get the second derivative of the Eq. (16), that is

(17) $$\begin{array}{c} \frac{d^{2}y}{dx^{2}}=\frac{12\left(1-\frac{a}{b}\right)y_{i}}{\left(1-2k\right)b^{4}}x^{2}-\frac{12\left(1-\frac{a}{b}\right)\cdot y_{i}\left(1+k\right)}{\left(1-2k\right)b^{3}}x+\frac{12\left(1-\frac{a}{b}\right)\cdot y_{i}k}{\left(1-2k\right)b^{2}}\\ =\frac{12\left(1-\frac{a}{b}\right)}{\left(1-2k\right)b^{2}}\left(\frac{x^{2}}{b^{2}}-\frac{1+k}{b}x+k\right)y_{i} \end{array}$$

Let that

$$\sigma_{0}=\frac{P}{bt}$$

Based on the relationship between the curvature of the ILCs and the transverse stresses, we get that [2,3]

(18) $$\begin{array}{c} \sigma_{T}\left(x\right)=\int_{0}^{b/2}\frac{d^{2}y}{dx^{2}}\sigma_{0}dy_{i}=\frac{12\left(1-\frac{a}{b}\right)P}{\left(1-2k\right)\cdot b^{2}tb}\left(\frac{x^{2}}{b^{2}}-\frac{1+k}{b}x+k\right){\int_{0}^{b/2}y_{i}dy_{i}\cdot \left(y^{2}\right|}_{0}^{b}\\ =\frac{3\left(1-\frac{a}{b}\right)P}{2\left(1-2k\right)\cdot b^{3}t}\left(x^{2}-b\left(1+k\right)x+kb^{2}\right) \end{array}$$

### Derivation of the splitting force and its location

By integrating the transverse tensile stresses along the axis of the struts, the total transverse force below the axis of the struts can be determined by

(19) $$\begin{array}{c} T_{b}=\int_{k\cdot b}^{b}\sigma_{T}tdx\\ =\int_{k\cdot b}^{b}\frac{3\left(b-a\right)P}{2\left(1-2k\right)\cdot b^{4}t}\left(x^{2}-b\left(1+k\right)x+k\cdot b^{2}\right)tdx\\ =\frac{3\left(b-a\right)P}{2\left(1-2k\right)\cdot b^{4}}\left[\frac{1}{3}\left(b^{3}-k^{3}b^{3}\right)-\frac{1}{2}b\left(k+1\right)\left(b^{2}-k^{2}b^{2}\right)+kb^{2}\left(b-kb\right)\right]\\ =\frac{3\left(b-a\right)P}{2\left(1-2k\right)\cdot b^{4}}\left[\frac{1}{3}b^{3}\left(1-k^{3}\right)-\frac{1}{2}b^{3}\left(k+1\right)\left(1-k^{2}\right)+kb^{3}\left(1-k\right)\right]\\ =\frac{3\left(b-a\right)P}{2\left(1-2k\right)\cdot b^{4}}\left[\frac{1}{3}b^{3}\left(1-k\right)\left(1+k+k^{2}\right)-\frac{1}{2}b^{3}{\left(k+1\right)}^{2}\left(1-k\right)+kb^{3}\left(1-k\right)\right]\\ =\frac{3\left(b-a\right)P}{2\left(1-2k\right)\cdot b^{4}}\left[b^{3}\left(1-k\right)\left(\frac{1+k+k^{2}}{3}-\frac{{\left(k+1\right)}^{2}}{2}+k\right)\right]\\ =-\frac{3\left(b-a\right)P}{2\left(1-2k\right)\cdot b^{4}}\left[\frac{{\left(1-k\right)}^{3}b^{3}}{6}\right] \end{array}$$

The location of the splitting force *d_b_* can be expressed as follows:

(20) $$d_{b}=\frac{\int_{k\cdot b}^{b}x\sigma_{T}tdx}{\int_{k\cdot b}^{b}\sigma_{T}tdx}$$

(21) $$\begin{array}{c} \int_{k\cdot b}^{b}x\sigma_{T}tdx\\ =\int_{k\cdot b}^{b}x\frac{3\left(b-a\right)P}{2\left(1-2k\right)\cdot b^{4}t}\left(x^{2}-b\left(1+k\right)x+k\cdot b^{2}\right)tdx\\ =\frac{3\left(b-a\right)P}{2\left(1-2k\right)\cdot b^{4}}\int_{k\cdot b}^{b}\left[x^{3}-b\left(1+k\right)x^{2}+k\cdot b^{2}x\right]dx\\ =\frac{3\left(b-a\right)P}{2\left(1-2k\right)\cdot b^{4}}\left[\frac{1}{4}\left(b^{4}-k^{4}b^{4}\right)-\frac{1}{3}b\left(1+k\right)\left(b^{3}-k^{3}b^{3}\right)+\frac{1}{2}kb^{2}\left(b^{2}-k^{2}b^{2}\right)\right]\\ =\frac{3\left(b-a\right)P}{2\left(1-2k\right)\cdot b^{4}}\cdot \frac{b^{4}\left(1+k\right){\left(k-1\right)}^{3}}{12}=\frac{\left(b-a\right)P\left(1+k\right){\left(k-1\right)}^{3}}{8\left(1-2k\right)} \end{array}$$

By Substituting Eqs. (19) and (21) into Eq. (20), then we can derive as:

(22) $$d_{b}=\frac{\int_{k\cdot b}^{b}x\sigma_{T}tdx}{\int_{k\cdot b}^{b}\sigma_{T}tdx}=\frac{\frac{\left(b-a\right)\left(1+k\right){\left(k-1\right)}^{3}P}{8\left(1-2k\right)}}{-\frac{{\left(1-k\right)}^{3}P}{4\left(1-2k\right)}\left(1-\frac{a}{b}\right)}=\frac{b}{2}\left(1+k\right)$$

## Conflicts of interest

None.
